# Therapeutic Use of Extraembryonic-Derived Tissues

**DOI:** 10.1155/2018/6082698

**Published:** 2018-06-13

**Authors:** Michael Uhlin, Mohamed Abumaree, Essam M. Abdelalim

**Affiliations:** ^1^Department of Clinical Science, Intervention and Technology, Karolinska Institutet, Stockholm, Sweden; ^2^Department of Clinical Immunology and Transfusion medicine, Karolinska University Hospital, Stockholm, Sweden; ^3^Department of Applied Physics, Royal Institute of Technology, Stockholm, Sweden; ^4^Stem Cells and Regenerative Medicine Department, King Abdullah International Medical Research Center, King Abdulaziz Medical City, and Ministry of National Guard Health Affairs, P.O. Box 22490, Riyadh 11426, Saudi Arabia; ^5^College of Science and Health Professions, King Saud Bin Abdulaziz University for Health Sciences, King Abdulaziz Medical City, and Ministry of National Guard Health Affairs, P.O. Box 3660, Riyadh 11481, Saudi Arabia; ^6^Diabetes Research Center, Qatar Biomedical Research Institute, Hamad Bin Khalifa University, Qatar Foundation, Education City, Doha, Qatar

Instead as being seen as medical waste, umbilical cord blood (UCB), placenta-derived cells, and other extraembryonic tissue are increasingly accepted as a high-quality source of cells for therapeutic use. The best-known application is the transplantation of hematopoietic stem cells (HSCT), while UCB has become an increasingly important graft source since UCB transplantation (UCBT) has been implemented in the last 3 decades. Recently, UCB, placenta, and extraembryonic-derived cells and tissues have been also investigated as a source for adoptive cell therapy.

The nonhematopoietic stem cell types in UCB as well as placenta-derived and extraembryonic cells and tissues include several types that can be used therapeutically and are readily expanded to sufficient numbers using established methods. Most notable of these are mesenchymal stromal cells (MSCs) and endothelial-like vascular progenitors (EPCs). To complicate it further, MSCs from different sources of the placenta seem to have very different properties.

To even further potentiate the use of extraembryonic-derived tissues for therapy, the sources have to be elaborately characterized. In this special edition, the potential use for this kind of tissues in this was highlighted in several cases illustrating its role in future regenerative medicine. Examples of papers are published in this special edition.

Endometriosis is characterized by the growth of the endometrium outside the uterus, mainly in the pelvic cavity. The pathophysiology of the endometriosis is still not completely understood. Previous reports suggested that there are several factors contributing to the pathogenesis of endometriosis, such as decreased immunosurveillance in the pelvic cavity and stem cells. There is a stem cell theory assuming that because of the retrograde menstruation, mesenchymal stromal cells (MSCs) present ectopically in the pelvic cavity. In this special issue, A. Fawaz et al. published a manuscript in which they provide characterization of the functional phenotype of MSCs in ectopic and eutopic endometria isolated from women with endometriosis. They examined whether the stromal cells of endometriotic ovarian cysts (ESCcyst) and endometrium (ESCendo) have a MSC phenotype. Interestingly, they showed that stromal cells from both ESCcyst and ESCendo have MSC characteristics and were able to differentiate into other cells, such as adipocytes and osteoblasts. It has been reported that MSCs have an immunosuppressive phenotype and express immunosuppressive molecules under increased inflammatory conditions. However, under low level of inflammation, they have an immunostimulatory phenotype and express high levels of proinflammatory cytokines. A. Fawaz et al. found that ESCcyst have more immunosuppressive characteristics than does ESCendo. Results published in this article suggested that the ESCcyst-immunosuppressive phenotype enhances immunosuppressive M2 macrophage, leading to a reduction in the immunosurveillance of ectopic lesions enhancing the growth of ESCcyst. This data supports the stem cell theory and the retrograde menstruation. In this special issue, another article has been published by the same group. In this article, they investigated the influence of allogeneic MSCs on cells isolated from endometriosis *in vitro*. Allogeneic MSCs were isolated from adipose tissue (Ad-MSC), and stromal cells were isolated from ESCendo and ESCcyst from women with endometriosis. They showed that Ad-MSCs enhance the proliferation, survival, and adhesion of ESC. Furthermore, ESCcyst migration was increased by Ad-MSCs. This article recommends that allogeneic Ad-MSCs are not suitable for therapeutic purposes of the endometriosis with ESCcyst, because they increase the growth and survival of ectopic endometrial tissue.

The placenta is rich with different types of cells and extracts that can be used for therapeutic approaches. In this special issue, O. Pogozhykh et al. published an interesting review article summarizing the types of placental derivatives and their applications in regenerative medicine. These types include cord blood cells, placental extracts, cord blood serum, isolated placental cells, amniotic and chorionic membranes, placental tissues, and amniotic fluid. The review provides information about the biobanking of placental components. Detailed information about the current clinical trial using placental derivatives is described in this review.

A Marmotti et al. described a method for isolation and expansion of UC-derived MSCs that were later successfully differentiated into chondrocytes. Their results would in the future potentially open the possibility to deliver an on-demand allogeneic population of cells for cartilage repair and bone regeneration.

